# Potential Role of Copper in Diabetes and Diabetic Kidney Disease

**DOI:** 10.3390/metabo13010017

**Published:** 2022-12-22

**Authors:** Guido Gembillo, Vincenzo Labbozzetta, Alfio Edoardo Giuffrida, Luigi Peritore, Vincenzo Calabrese, Claudia Spinella, Maria Rita Stancanelli, Eugenia Spallino, Luca Visconti, Domenico Santoro

**Affiliations:** 1 Unit of Nephrology and Dialysis, Department of Clinical and Experimental Medicine, University of Messina, 98125 Messina, Italy; 2 Department of Biomedical and Dental Sciences and Morpho-Functional Imaging, University of Messina, 98125 Messina, Italy; 3 Unit of Nephrology and Dialysis, Ospedali Riuniti Villa Sofia Cervello, University of Palermo, 90146 Palermo, Italy

**Keywords:** Diabetic Kidney Disease, copper, diabetes, zinc, diabetic nephropathy, chronic kidney disease, gestational diabetes mellitus

## Abstract

Copper is a fundamental element for the homeostasis of the body. It is the third most abundant essential transition metal in humans. Changes in the concentration of copper in the blood are responsible for numerous diseases affecting various organs, including the heart, brain, kidneys, and liver. Even small copper deficiencies can lead to the development and progression of several pathologies. On the other hand, excessive exposure to copper can cause toxicity in many human organs, leading to various systemic alterations. In the kidney, increased copper concentration in the blood can cause deposition of this element in the kidneys, leading to nephrotoxicity. One of the most interesting aspects of copper balance is its influence on diabetes and the progression of its complications, such as Diabetic Kidney Disease (DKD). Several studies have shown a close relationship between copper serum levels and altered glycemic control. An imbalance of copper can lead to the progression of diabetes-related complications and impaired antioxidant homeostasis. A high Zinc/Copper (Zn/Cu) ratio is associated with improved renal function and reduced risk of poor glycemic control in patients with type two diabetes mellitus (T2DM). Furthermore, the progression of DKD appears to be related to the extent of urinary copper excretion, while regulation of adequate serum copper concentration appears to prevent and treat DKD. The aim of this review is to evaluate the possible role of copper in DKD patients.

## 1. Introduction

Copper (Cu) is an essential trace metal that is the third most abundant essential transition metal in humans. The mains sources of copper are foods such as vegetables, cereals, meat, fish, poultry, and legumes [1]. The average daily intake of copper is between 1 and 1.6 mg, with the recommended dose for adults being 900 mcg/day [2]. Copper is present in the body in concentrations of at least 50 mg to a maximum of 120 mg. This microelement is found in high concentrations in the liver, brain, and bones, and to a lesser extent in the heart, pancreas, and kidneys [3,4] (Table 1).

Copper is absorbed in the stomach and proximal small intestine, and is favored by the acidic environment that dissociates copper from dietary macromolecules [6]. The absorption process occurs by active transport processes when the daily copper intake is low, whereas it occurs by passive diffusion when the intake is high. The ingested copper binds to albumin and plasma amino acids and is transported to the liver where it is internalized. At this point, ceruloplasmin present in the liver binds the copper and transports it to peripheral tissues. The transporter binds to the cell surface and releases the transported copper. The liver also produces metallothionein, which can serve as a storage protein.

Its concentration in the body depends on the balance between absorption in the small intestine and excretion via the liver with bile [7].

Copper plays an important role in the regulation of numerous enzymes and the synthesis of structural components and is involved in many physiological pathways and biological processes including angiogenesis, response to hypoxia, and neuromodulation [8].

A key role in preventing copper deficiency or toxicity is played by the P-type Wilson ATPase, which is responsible for transporting copper from the liver into the secretory pathway (about 50% of copper is excreted via bile, the rest via other gastrointestinal secretions) [9]. Mutations of this gene lead to a lack of copper transport from the liver into the bile and to a deficient incorporation of copper into ceruloplasmin. Other copper-containing enzymes are: Zinc-Cu superoxide dismutase, which plays a fundamental role in oxidative processes; dopamine mono-oxygenase, which is involved in the synthesis of neurotransmitters; lysyl oxidase, which is involved in bone formation; Leiden factor V, the deficiency of which leads to coagulation disorders; cytochrome C oxidase, the deficiency of which can manifest itself through several systemic symptoms [10].

Copper deficiency is characterized by hair and skin changes, muscle weakness, neurological disorders such as ataxia, neuropathy and cognitive impairment, edema, hepatosplenomegaly, and osteoporosis. It can also lead to anemia and neutropenia, the main hematologic features of copper deficiency.

In addition, copper is involved in processes that regulate oxidative stress (OS). Under physiological conditions, there is a balance between the products of metabolic processes that use oxygen (O2) as fuel for energy production, the so-called reactive oxygen species (ROS) and antioxidant agents. When this balance is disturbed, an increase in circulating ROS leads to the phenomenon of OS, which can cause damage to several cellular structures. If not adequately controlled, OS may be involved in the development of chronic and/or degenerative diseases such as cancer and cardiovascular disease [11]. In addition, minor copper deficiencies may contribute to the onset and progression of several pathologies, including diabetes. On the other hand, excessive copper concentration in the body can cause toxicity in many human organs, resulting in various diseases and, in rare cases, death.

Diabetes and Diabetic Kidney Disease (DKD) represent a real pandemic problem both for the public economy and for global health [12]. The development of novel therapies has helped to counteract this global phenomenon and ensure a more personalized approach, but dietary regulation and the adequate intake of essential elements are an indispensable aspect of treatment strategies [13,14].

In the kidney, a correct balance of copper seems to be essential: an increased blood concentration of this ion in the kidney may condition its renal deposition, leading to nephrotoxicity associated with interstitial damage that can lead to progressive renal function impairment [15]. Copper excretion in the urine may be related to dissociation from the albumin–copper complex of the serum as it passes through the kidney. In diabetics with progressive renal dysfunction, urinary excretion of this element may be due to dissociations of both albumin–copper and ceruloplasmin–copper complexes filtering through the damaged glomerulus. Urinary copper overload of the altered renal tubules may play a role in the progression of renal dysfunction in patients with advanced CKD.

Previous studies have shown that hypercupremia is associated with the development of Chronic Kidney Disease (CKD) [16]. On the contrary, a reduction in the renal filtration rate leads to impaired renal excretion of copper and consequently to increased blood concentrations with corresponding potential complications [17].

The Mendelian randomization study by Ahmad et al. reports that genetically determined elevated circulating copper levels may be a causal risk factor for CKD and could possibly reduce estimated glomerular filtration rate (eGFR) and rapidly declining renal function [18]. A cross-sectional study of 3553 adults from Hunan, China, found that copper in urine is a risk factor for impaired kidney function [19]. Guo et al. demonstrated that whole blood copper levels were remarkably related to CKD risk and showed a positive dose–response relationship in the elderly Chinese population [20]. However, in another nested case-control study of 350 adolescents in northwestern Nicaragua, urinary Cu levels were found to have no significant association with loss of renal function in participants at risk for CKD of unclear etiology [21].

All of these findings show that the interaction between copper and kidney disease goes both ways, as imbalances in the homeostasis of circulating copper levels can also be associated with altered renal excretion and changes in protein metabolism in patients with CKD and with disease progression. The aim of our review was to evaluate the role of copper in urine and serum in diabetic patients with renal involvement.

## 2. Role of Copper on DM and DKD: Animal Studies

Diabetes and diabetes-related complications are associated with dysregulation of fundamental elements of homeostasis. Over the years, diabetes has been shown to affect copper metabolism and, moreover, the antioxidant activity of copper containing enzymes.

Uriu-Adams et al. [22] investigated the role of copper in mice by inducing diabetes through the administration of streptozocin. After five weeks of treatment with a low copper diet, the animals were killed and analyzed at different time points. It was found that copper metabolism was impaired in diabetic rats with a low activity of copper-zinc superoxide dismutase (SOD), and that this was even more reduced on a low copper diet. Conversely, plasma concentrations of metallothioin and ceruloplasmin in the liver and kidneys were elevated in diabetic rats compared to control rats. These metabolic changes appear to be caused by increased OS and inflammation which in turn can lead to the progression of diabetic complications.

In a study by Gòmez T et al. [23], the concentrations of elements such as zinc, copper, iron, calcium and magnesium were examined in a group of 10 mice that became diabetic as a result of streptozocin (STZ) administration (100 mg/kg), compared to a control group (*n* = 10). It was found that the concentrations in serum and in liver and kidney tissues did not differ significantly between the two groups. Urine concentrations were lower in the diabetic mouse group (*p* < 0.05) than in the control group, but not when calculated on the basis of 24-h urine. In this case, the concentrations in the urine of the diabetic mice were higher (*p* < 0.01). This indicates that urine excretion was strongly correlated with the amount of urine collected in 24 h (r > 0.7; *p* < 0.001). According to the authors, the changes in mineral metabolism in the diabetic mice could be due to the endocrine imbalance caused by the disease.

OS is also involved in the pathogenesis of T2DM through the production of reactive oxygen species (ROS), and it is known that the production of ROS increases in the presence of copper ions through the Fenton reaction. The aim of the study by Tanaka et al. [24] was to evaluate the role of copper in the development of T2DM and the effect of a copper chelating agent in the treatment of T2DM. In diabetic C57BL/KsJ-db/db mice, copper levels were significantly higher than in non-diabetic mice. The diabetic db/db mice were treated with a copper chelating agent, tetratiomolybdate. Both serum copper levels and serum levels of ROS were significantly reduced by the chelating agent in the diabetic mice to levels comparable to those in non-diabetic mice. The chelating agent has been shown to effectively reduce insulin resistance and improve glucose intolerance in recessive homozygous diabetic mice.

Several animal studies have examined the activity of some proteins associated with DKD and whether it improves after treatment with copper chelators such as thethylenetetramine (TETA). Lu J et al. [25] investigated treatment with selective copper chelators, such as TETA and other less selective options, and the effects on slowing the onset of DM-related complications and/or suppression of OS triggered by DM. One group of mice that became diabetic from STZ administration (55 mg/kg i.v.) was treated with oral TETA for 8 weeks, starting at 8 weeks after the onset of diabetes. A second group was treated with one of the less selective chelating agents such as D-penicillamine, deferiprone, or Zn-acetate. The diabetes had damaged cardiac and renal function in the first 8 weeks after induction by decreasing the ability of the heart to respond to increased afterload and significantly increasing the ratio of albumin/creatinine in the urine, while also decreasing the total antioxidant power and heparan sulfate content in cardiac and arterial tissue and increasing its renal excretion. It was observed that cardiac and renal function and extracellular vessel activity SOD significantly improved in the TETA-treated diabetic mice compared to the control group treated with the less selective chelator.

Attention was focused on changes in renal copper homeostasis and how its regulation may influence protection against the pathogenic mechanisms that cause DKD. The response to TETA in animals with DKD was investigated in the study of Gong D et al. [26]. Specifically, after an 8-week induction of diabetes with streptozocin, rats were treated with oral TETA (34 mg/day orally) for an additional 8 weeks and compared to untreated diabetic rats. At 8 weeks after TETA administration, a marked improvement in urinary albumin/creatinine ratio, a reduction in collagen levels, and normalization of TGF-beta1, homologous MAD (SMAD) 4, phosphorylated SMAD2, fibronectin-1, collagen-III, and collagen were observed, in addition to IV, inhibitor of plasminogen activator-1, and semicarbazide-sensitive amine oxidase in the renal cortex. These data confirm the ability of copper chelation to treat early stage DKD.

In the study by Gong D. et al. [27], they used diabetic and non-diabetic rats, as well as diabetic rats treated with TETA, to evaluate and upregulate tubulointerstitial nephritis antigen (TINag), selective voltage-dependent anion channel (VDAC) 1, and VDAC2; in contrast, mitochondrial HSP 60, Cu/Zn-SOD, glutathione S-transferase alfa3, and aquaporin-1 were downregulated. It was observed that after treatment with TETA, albuminuria levels improved and the activity of D-amino acid oxidase-1, epoxide hydrolase-1, aquaporin-1, and several mitochondrial proteins as well as TINag in diabetic rats, collagen VIalpha1, actinin 4alpha, factor 1 inducing apoptosis, cytochrome C, and histone H3 normalized. The results highlight that this type of therapy could be useful for the treatment of DKD.

Alterations in copper metabolism appear to have teratogenic effects in diabetic rats. In the study by Jankowski et al. [28], diabetic and non-diabetic mice were compared with a high copper (12 micrograms/g) and a low copper (1 microgram/g) diet started two weeks before mating and continued throughout gestation. Copper chelators were used in mice fed a low-copper diet to further reduce serum copper levels. Pregnancy was terminated on day 20 of gestation. In the low copper diet groups, low levels of copper were found in the liver of the fetuses, regardless if they were diabetic or not. It was also found that maternal copper intake did not cause structural and skeletal abnormalities in the fetuses more often than in the low-copper diet group. In addition, in all groups, the activity of SOD tended to be reduced when they were on a low copper diet.

In a study by G K Saunders [29], 10 mice in which diabetes had been induced by the administration of streptozocin were compared with 5 healthy mice. Five of the diabetic mice were treated with penicillamine to chelate the copper. Glomerular Filtration Rate (GFR) and maximal tubular concentration (Tm) for sodium p-aminoippurate (PAH), glucose, and inulin were calculated and compared with copper levels using the Student t test. Copper levels in the chelator-treated diabetic mice decreased significantly (*p* < 0.01), indicating that penicillamine effectively removes copper from the diabetic kidney. The diabetic mice had increased GFR and increased Tm for glucose and PAH. However, the group of diabetic mice treated with the copper chelator did not differ from the untreated diabetic mice, suggesting that excess renal copper does not affect kidney function in the early stages of diabetes (Table 2).

## 3. Copper in T1DM Patients

OS is favored by hyperglycemia, serum levels of heavy metals, and their binding proteins. Among these ions, copper is also involved in the pathogenesis of systemic diseases such as Type 1 Diabetes Mellitus (T1DM).

Squitti R et al. [30] aimed to assess serum levels of total copper, the copper-containing protein ceruloplasmin, unbound copper to ceruloplasmin, and other metals in 63 subjects with T1DM compared to a group of 65 healthy subjects.

The most important finding of this study was the higher copper and ceruloplasmin levels in people with T1DM compared to control subjects. An increase in copper levels was the strongest factor associated with T1DM, with the risk of developing the disease increasing about 15-fold in standard deviation. According to these results, T1DM appears to be associated with a change in serum levels of several metals, particularly copper.

In line with this thesis, Viktorinova et al. [31] observed that alteration in the metabolism of elements such as copper, zinc, and magnesium may contribute to the progression of diabetes. Plasma levels of these ions were studied in patients with Diabetes Mellitus (DM) and in healthy controls. The link between glycated hemoglobin and metal levels was also studied. Thirty-six patients with DM (11 with T1DM and 25 with type 2 diabetes mellitus (T2DM) were recruited and compared with 34 healthy subjects, matched for age, sex, and duration of diabetes.

Patients with DM were found to have higher copper levels (*p* < 0.001) and a higher Cu/Zn ratio (*p* < 0.0001) with Zinc (Zn) levels (*p* < 0.01) and Magnesium (Mg) levels (*p* < 0.0001) compared to the control group. A negative correlation was present between Cu and Zn (r = −0.626, *p* < 0.0001) in patients with DM. Glycated hemoglobin levels correlated directly.

With Cu levels (r = 0.709, *p* < 0.001) and Cu/Zn ratio (r = 0.777, *p* < 0.001) and inversely with Zn (r = −0.684, *p* < 0.001) and Mg (r = −0.646, *p* < 0.001).

Zargar AH et al. [32] specifically investigated the concentrations of copper, zinc, and magnesium in patients with T1DM, as they suspected that these elements may play a role in the pathogenesis and progression of the disease. They recruited 37 patients with T1DM and 25 healthy non-diabetics. DM patients and healthy volunteers had comparable mean plasma concentrations of copper and magnesium. Plasma zinc levels were significantly higher (*p* = 0.022) in DM patients (17.78 ± 0.6 micromol/L) than in control subjects (15.80 ± 0.75 micromol/L), while glycemic control and the presence of microalbuminuria had no effects on plasma levels of copper, zinc, and magnesium. These results show that plasma levels of copper and magnesium were not significantly altered in patients with T1DM compared to controls, in contrast to zinc levels.

These data seem to suggest that the metabolism of these ions may contribute to the progression and development of T1DM and its complications, but the results are still controversial (Table 3).

## 4. Copper in T2DM Patients

Copper seems to exert an influence not only in T1DM patients but also in T2DM subjects. The relationship between glycemic control and blood copper levels has been investigated in several studies on patients with T2DM.

In the study by Raudenska M et al. [33], 70 subjects with T2DM and 80 healthy controls were recruited and compared by examining the anti-radical capacity, levels of metallothioneins, zinc, copper and selected antioxidants such as bilirubin, cysteine, and glutathione. The results showed that anti-radical capacity, conjugated bilirubin, and copper were significantly higher in diabetics, while metallothioneins and glutathione decreased.

In their study, Naka T et al. [34] examined 132 patients with T2DM and measured their serum copper status. The levels of this ion correlated with the levels of Glycosylated hemoglobin (HbA1c) (r = 0.176, *p* = 0.044). In the three months after enrolment, it was investigated whether treatment and lowering HbA1c levels affected serum copper levels. Glycosylated hemoglobin was decreased (8.7% to 6.8%, *p* < 0.001) and copper levels tended to decrease (from 105.7 μg/dL to 101.8 μg/dL, *p* = 0.069). From the results obtained, it appears that the glycemic control in patients with T2DM can alter their serum copper levels.

Zargar et al. [35] also demonstrated a relationship between trace elements and diabetes. They studied 83 patients with non-insulin-dependent diabetes mellitus, diagnosed at 3.9 +/− 3.6 years of age, excluding patients with DKD, and 30 healthy controls using fasting glucose and fructosamine levels. Serum levels of copper, zinc, and magnesium were analyzed. Only copper levels were significantly increased in diabetic patients (*p* < 0.01), without factors such as age, sex, and duration of diabetes influencing the concentrations of the trace elements. These results are in partial contrast to those of the cross-sectional study by Sonkar et al. [36]. The authors conducted the study on 150 patients with T2DM and 50 control subjects and showed how blood glucose levels can be influenced by serum concentrations of trace elements. Serum levels of zinc, copper, chromium, selenium, and magnesium were measured with a colorimetric kit, while fasting blood glucose and glycated hemoglobin were determined with standard tests. Of these 150 diabetics, 128 had glycemia that was not well controlled with a glycated hemoglobin >7. Patients also had comorbidities such as hypertension and hypothyroidism. Serum levels of zinc, copper, selenium, and magnesium were significantly lower in patients with T2DM than in controls (62.89 vs. 74.95 μg/dL, *p* < 0.05; 116.30 vs. 150.39 μg/dL, *p* < 0.001; 8.57 vs. *p* < 0.05; 16.16 μg/dL, *p* < 0.001; 1.92 vs. 2.31 mg/dL, *p* < 0.05, respectively).

A higher rate of T2DM was observed in Hispanics, and these populations appear to be more exposed to endocrine-disrupting chemicals, including some metals and metalloids. In their study, Weiss C et al. [37] wanted to determine how exposure to metals was related to disease progression. Eight urinary metal concentrations were examined in relation to the glycemic status in 414 normoglycemic and prediabetic adults. Multivariate linear regression was used to quantify differences in pancreatic beta cell function, insulin resistance, and insulin sensitivity (defined respectively by the acronyms HOMA-β, HOMA-IR, HOMA-S), the amount of plasma insulin, plasma glucose, and Hb1Ac, which is associated with increased urinary metal concentrations in the urine. Arsenic and molybdenum in urine were associated with lower pancreatic beta cell function, insulin resistance, lower plasma insulin levels, and higher insulin sensitivity. In addition, higher urinary copper levels were associated with decreased beta cell function; higher concentrations of the eight metal mixtures were associated with lower HOMA-β levels, HOMA-IR, and plasma insulin, and higher HOMA-S levels. These data suggest that arsenic, molybdenum, and copper in particular are associated with changes in glucose homeostasis even in the non-diabetic population (Table 4).

## 5. Role of Copper in Diabetic Kidney Disease

Renal failure and diabetes are also associated with disturbances in antioxidant homeostasis and chronic inflammation.

The study by Stancic A et al. [38] compared the activity of copper-zinc SOD in diabetic hypertensive patients with or without renal insufficiency and a control group. The results showed that SOD activity was significantly higher in diabetics with renal insufficiency, suggesting that disturbances in antioxidant homeostasis are associated with complications of diabetes such as hypertension and renal failure.

**Figure 1:**
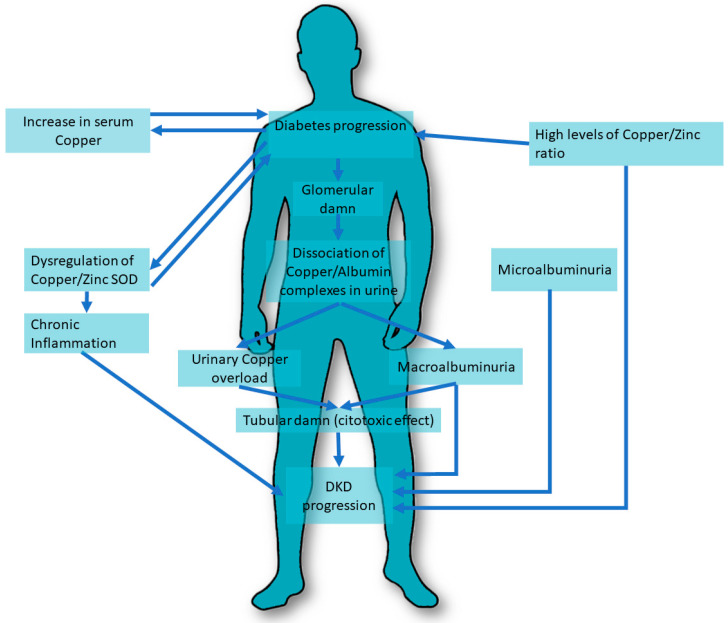
Copper pathways involved in DKD. DKD, Diabetic Kidney Disease. SOD, superoxide dismutase.

The extent of copper excretion in urine was associated with the different stages of DKD. In studies by Ito S. et al. [39], 41 type 2 diabetic patients with different stages of nephropathy and 10 healthy controls were recruited and serum copper/albumin and copper/ceruloplasmin ratios were determined and tested whether they tended to dissociate in response to changes in urine pH. The results showed that urinary copper was significantly increased only in patients with macroalbuminuria. Urinary copper/ceruloplasmin and copper/albumin ratios were greater than in serum and equal between patients and healthy controls, except for the copper/albumin ratio in patients with macroalbuminuria. Reports in urine decreased when nephropathy worsened. Copper tends to dissociate from its carrier protein under acidic pH conditions. A damaged glomerulus due to nephropathy may cause greater dissociation of the copper/albumin and copper/ceruloplasmin complexes, and urinary copper overload may, in turn, play an important role in the progression of nephropathy (Figure 1).

Copper is essential to ensure cardiovascular wellbeing. Several studies have shown that a deficiency of this ion may be a risk factor for the development of cardiovascular disease, especially in patients with T2DM with and without DKD. An example of this is the study by Al-Bayati et al. [40], which compared 55 patients with type 2 diabetes divided into two subgroups (the first group *n* = 31 with microalbuminuria between 30 and 299 μg/mg and a second group *n* = 29) with an albumin level below 30 μg/mg with 37 healthy subjects. The data showed an increase in urinary copper excretion in the group with microalbuminuria associated with a decrease in antioxidant enzymes compared to the control group, *p* < 0.05.

Talaei et al. [41] studied urinary copper levels in T2DM patients with microalbuminuria compared to patients without albuminuria by examining 42 patients with DKD and comparing them to a group of 40 healthy subjects. The 24h urinary copper levels were 36.14 μcg/L (14.54–57.74) and 14.77 μcg/L (10.17–19.37) in the case and control groups, respectively (*p* = 0.003). Diabetics with microalbuminuria appeared to have a greater urinary excretion in the 24 h, although a toxic effect of this high excretion in the progression of DKD cannot be excluded.

An important mechanism to evaluate the copper homeostasis is its association with zinc levels. Zinc is fundamental for the function of the antioxidant enzyme copper–zinc SOD, and an appropriate balance between these two micronutrients is of pivotal importance to controlling inflammation and reducing risk factors associated with DKD [42].

Several authors have associated the Zn/Cu ratio with glycemic status, renal function, and metabolic parameters in patients with and without T2DM. Hamasaki et al. [43] conducted a cross-sectional study of 149 diabetic and 206 non-diabetic patients measuring the levels of Zn and Cu, their ratio, the prevalence of type 2 diabetes, and the degree of renal function. A high Zn/Cu ratio was associated with improved renal function scores (β = 0.137, *p* = 0.014) and a reduced risk of poor glycemic control in patients with type 2 diabetes, which was assessed by multivariate logistic regression analysis (HbA1c ≥ 7%) (odds ratio = 0.382; 95% confidence interval, 0.165–0.884; *p* = 0.025).

In another cross-sectional study conducted by Takao et al. [44], the authors analyzed data from the Asahi Diabetes Complications Study to assess the role of the copper/zinc ratio in the DKD population. These data showed that a higher value of this ratio was associated with a higher prevalence of renal involvement during the course of T2DM.

While several studies have demonstrated a possible association between urinary copper excretion and DKD progression, the results related to serum copper levels are still controversial. Serum copper concentration appears to be altered in T1DM and T2DM patients compared to controls, with and without DKD [8,9,15,45]. These data are in contrast to the data of Prabodh et al. [46], who showed that there are no differences in serum copper levels in the patients with DKD compared to a group of healthy subjects. A group of 40 DKD patients and 40 control subjects were compared and fasting glucose, post-meal glucose, glycated hemoglobin, microalbuminuria, copper, and magnesium levels were determined. The results showed that the mean concentrations of fasting and postprandial glycemia, glycated hemoglobin, and microalbuminuria were significantly higher in the patients than in the control group. Mean copper levels in the DKD group, 165.42 ± 5.71 μg/dL, showed no significant differences compared with controls, 166.6 ± 5.48 μg/dL, (*p*> 0.05). These results suggest that hypomagnesemia may be related to the development of DKD, and copper levels do not seem to play a prominent role in the development of DKD.

The available literature confirms a possible association between urinary copper excretion and DKD, while the role of serum levels of this ion needs further investigation. The copper/zinc ratio may be a useful biomarker for both DM and the treatment of DKD: the correct balance of these two ions seems to counteract inflammatory processes and to be associated with adequate metabolic homeostasis in the DM population [47] (Table 5).

## 6. Conclusions

Most of the studies reported in our review show an association between advanced age and higher copper levels in patients with diabetes, while in the younger population the risk of developing T1DM still increases 15-fold for one standard deviation of copper levels.

Age-related chronic diseases lead to mechanisms that increase serum concentrations of copper and decrease serum concentrations of zinc, especially in the presence of inflammatory conditions, thus a common feature of several age-related chronic diseases is an increase in the copper/zinc ratio [48,49]. The copper/zinc ratio is also associated with physical decline and mortality in the elderly population [50].

According to the available evidence, copper levels, diabetes, and its complications are not simply positively correlated, but have a complex relationship.

Nevertheless, more specifically designed prospective articles should be conducted to understand the dynamic relationship of copper, copper/zinc balance, and the progression of diabetes. These data indicate the need for dietary and health behavior changes to better control the potential complications associated with T1DM and T2DM. Measurement of trace elements is a supportive tool in the management of this pandemic disease.

People with DKD are more likely to have a disturbance in the homeostasis of essential metals. The imbalance of copper can lead to the progression of diabetes-related complications and impaired antioxidant homeostasis. A high Zn/Cu ratio seems to be associated with improved renal function and a lower risk of poor glycemic control in T2DM patients.

In addition, the extent of urinary copper excretion appears to be related to the DKD progression, which plays a possible role in the opposing DM complications.

Experimental models in vitro, as well as animal and human studies, suggest that maintaining adequate copper levels may play a role in the pathophysiology of diabetes and DKD. Unfortunately, there is a lack of large and interventional studies focusing on copper balance to confirm these findings. Evidence on the association between copper and DKD is still scarce and the results remain inconsistent. It should also be emphasized that the function of copper should be considered as part of a more complex system of factors that influence oxidative stress and condition its protective/damaging influence on DM and DKD. More studies are needed to adequately investigate the role of copper in DKD and the results available in the literature are still controversial.

## Figures and Tables

**Table 1:** Recommended Dietary Allowances (RDAs) for Copper [5].

Age	Male	Female	Pregnancy	Lactation
14–18 years	890 mcg	890 mcg	1000 mcg	1300 mcg
19+ years	900 mcg	900 mcg	1000 mcg	1300 mcg

**Table 2:** GFR, Glomerular Filtration Rate. SOD, Superoxide Dismutase. T2DM Type 2 Diabetes Mellitus, TETA, thethylenetetramine. UACR, Urine Albumin–Creatinine Ratio.

ANIMAL STUDIES	**Study, Year**	**Population**	**Control**	**Results**
	Saunders et al. [29] 1986	5 diabetic mice treated with penicillamine 5 diabetic mice untreated	5 non-diabetic mice	GFR and tubular maximum for both p-aminohippurate and glucose were increased in both diabetic groups than in control group *p* < 0.05 No difference between two diabetic groups
	Jankowski M A et al. [28] 1993 Case-Control Study	Diabetic and non-diabetic mice on a diet rich in copper (12 µg/g)	Diabetic and non-diabetic mice on a diet low in copper (1 µg/g)	Diabetic mice had fewer implantation sites, live births, and smaller fetuses with larger placentas than non-diabetic mice Gross external malformation was noted only in diabetic mice Maternal high copper intake did not cause fetal abnormalities more frequently than low copper intake.
	Uriu-Adams et al. [22] 2005 Case-Control Study	Diabetic mice, both with adequate and deficient diets in copper	Non-diabetic mice, both with adequate and deficient diet in copper	In diabetic rats, the metabolism of copper was impaired with a low activity of copper-zinc superoxide dismutase (SOD), and this was reduced even more with diets low in copper. The levels of plasma metallothioin and ceruloplasmin in the liver and the kidneys were elevated in diabetic rats compared to control rats.
	Gong et al. [26] 2008	Diabetic mice treated for 8 weeks with thethylene-tetramine	Diabetic mice untreated for diabetes	A marked improvement in the urinary albumin/creatinine ratio after TETA administration
	Lu J et al. [25] 2010 Case-Control Study	Diabetic mice treated for 8 weeks with thethylene-tetramine	Diabetic mice treated for 8 weeks with penicillamine, deferiprone, or Zn acetate	TETA treatment increased the resistance of cardiac output to the effects of increasing afterload pressure, while treatment with other chelators did not ameliorate cardiac function. TETA treatment significantly lowered the elevated uACR, while others chelators did not show any significant improvement.
	Gomez et al. [23] 2017 Case-Control Study	10 diabetic mice	10 non-diabetic mice	Serum (19.87 ± 0.89 vs. 17.08 ± 0.87), liver (40.44 ± 4.09 vs. 33.53 ± 3.98), and kidney tissue (64.15 ± 7.16 vs. 45.71 ± 2.60) concentrations did not differ significantly between diabetic and non-diabetic rats. Urinary concentrations were lower in the diabetic mice group (μmol/L 3.19 ± 0.45) than in the control group (4.71 ± 0.56) *p* = 0.050 Daily mineral excretion was higher in the diabetic mice group (0.10 ± 0.02) than in the control group (0.03 ± 0.01) *p* = 0.001
	Tanaka et al. [24] 2017 Case-Control Study	Diabetic mice	Non-diabetic Mice	Serum copper ion levels in diabetic mice tended to be higher compared to those in non-diabetic mice at 10 weeks of age (64.6 ± 15.1 µg/dL vs. 43.0 ± 3.8 µg/dL). After diabetic mice were treated with tetratiomolybdate 0.01 mg/mL or 0.02 mg/mL, serum copper levels were reduced to levels comparable to those of non-diabetic mice, 59.0 ± 17.4 and 46.2 ± 20.3 *p* < 0.05, respectively. Similarly, serum ROS levels were reduced, 44.8 ± 26.7 and 37.1 ± 11.7 *p* < 0.05

**Table 3:** Copper in T1DM patients. Cu, Copper; T1DM, Type 1 Diabetes Mellitus; T2DM, Type 2 Diabetes Mellitus; Zn, Zinc.

COPPER IN T1DM PATIENTS	**Study, Year**	**Population**	**Control**	**Results**
	Zargar et al [10] (2002) Cross-sectional study	37 patients with T1DM Age 21.78 ± 1.22 years	25 healthy subjects	No significant difference in Cu and Mg levels between patients and the control group Plasma Zn levels higher in patients (17.78 ± 0.6 μmol/L) vs. controls (15.80 ± 0.75) *p* value = 0.022
	Viktorìnovà et al [9] (2009) Cross-sectional study	11 patients with T1DM Age 49.9 ± 9.4 (37–63) years 25 patients with T2DM Age 49.9 ± 9.4 (37–63)	34 healthy subjects	Higher copper levels in patients (18.73 ± 2.6) vs. control (17.37 ± 2.4) *p* value < 0.001 Higher Cu:Zn Ratio in patients (1.42 ± 0.3) vs. control (1.21 ± 0.1) *p* value < 0.0001 Reduced levels of Zn in patients (13.48 ± 2.2) vs. control (14.41 ± 1.8) *p* value < 0.01 Reduced levels of Mg in patients (0.77 ± 0.2) vs. control (0.90 ± 0.1) *p* value < 0.0001 Positive correlation between plasma levels of HbA1c and Cu in patients r = 0.709 *p* < 0.001 Positive correlation between HbA1c and Cu/Zn ratio in patients r = 0.777 *p* < 0.001 Negative correlation between plasma levels of HbA1c and Zn in patients r = −0.684 *p* < 0.001 Negative correlation between plasma levels of HbA1c and Mg in patients r = −0.646 *p* < 0.001
	Squitti et al [8] (2019) Cross-sectional study	63 patients With T1DM Age 40.4 ± 15.3 years	65 healthy subjects	Higher copper levels in patients (17.9 ± 4.8 (μmol/L) vs. control (14.1 ± 3.8 (μmol/L). *p* value < 0.0001 Higher ceruloplasmin levels in patients (30.1 ± 9.5) vs. control (24.4 ± 6.4) *p* value < 0.0001 Higher Cu:Zn Ratio in patients (1.3 ± 0.5) vs. control (0.9 ± 0.3) *p* value < 0.0001 A 15-fold increase of risk of developing T1DM for a standard-deviation increas in copper levels

**Table 4:** Copper in T2DM patients. Hb, Hemoglobin. T2DM; Type 2 Diabetes Mellitus.

COPPER IN T2DM PATIENTS	**Study, Year**	**Population**	**Control**	**Results**
	Zargar et al. [35] 1998 Cross-sectional Study	83 patients With T2DM Age 50.7 ± 8.47 years	30 healthy subjects	Higher serum copper levels in patients group (16.87 + 4.69) vs. control group (13.91 ± 3.02) *p* < 0.01 Higher serum zinc levels in patients group (17.19 + 4.92) vs. control group (15.80 ± 4.12) *p* < 0.01
	Raudenska et al. [33] 2013 Cross-sectional Study	70 patients With T2DM Age 69 (54–84)years	80 healthy subjects Age 52 (45–64) years	Higher copper levels in patients (1.8 [1.4–2.9]) vs. control (1.5 [1.1–2.3]) *p* = 0.0018 Higher coniugated bilirubin levels in patients (4.8 [3.5–7.2]) vs. control (3.3 [2.7–3.5]) *p* < 0.0001 Reduced Metallothionins levels in patients (0.8 [0.7–0.9]) vs. control (0.9 [0.8–1.3]) *p* < 0.0001 Reduced glutathione levels in patients [912 (754–1017)] vs. control [1646 (966–2695)] *p* < 0.0001
	Naka et al. [34] 2013 Observational Study 3 months of follow up	132 patients With T2DM Age 58.5 ± 12.5 years	No control group	Positive correlation between serum copper and HbA1c levels r = 0.176, *p* = 0.044. Serum copper levels were significantly associated with HbA1c (r = 0.18, *p* < 0.05), Hb (r= −0.170, *p* = 0.051), and creatinine (r= −0.170, *p* = 0.051). HbA1c (β = 0.224, *p* = 0.011) and Hb (β= −0.220, *p* = 0.013) were independent determinants of serum copper levels. Serum ceruloplasmin or urinal copper levels were not associated with HbA1c levels (*n* = 53, r= −0.074, *p* = 0.60 and *n* = 103, r = 0.031, *p* = 0.757, respectively). After 3 months glycemic control, As HbA1c levels were decreased (from 8.7% to 6.8%, *p* < 0.001), copper levels tended to be decreased (from 105.7 µg/dL to 101.8 µg/dL, *p* = 0.069)
	Sonkar et al. [36] 2021 Cross-sectional Study	150 patients With T2DM Age 52.5 (9.69) years	50 healthy subjects	Lower serum copper levels in patients group (µg/dL 116.30) vs. control group (µg/dL 150.39) *p* < 0.001 Lower serum zinc levels in patients group (µg/dL 62.89) vs. control group (µg/dL 74.95) *p* = 0.0091 Lower serum selenium levels in patients group (µg/dL 8.57) vs. control group (µg/dL 16.16) *p* < 0.001 Lower serum magnesium levels in patients group (mg/dL 1.92) vs. control group (2.31) *p* = 0.046

**Table 5:** Copper in Diabetic Kidney Disease. Cu, Copper. SOD, Superoxide Dismutase. T2DM, Type 2 Diabetes Mellitus. Zn, Zinc.

DIABETIC KIDNEY DISEASE	**Study, Year**	**Population**	**Control**	**Results**
	Ito et al. [39] 2001 Case-Control Study	Group I: 15 diabetic patients with normoalbuminuria Age 60 ± 7 Group II: 14 diabetic patients with microalbuminuria Age 61 ± 9 Group III: 12 patients with macroalbuminuria Age 66 ± 8	10 healthy subjects Age 56 ± 10	Serum copper levels, serum ceruloplasmin levels, and serum copper/ceruloplasmin ratio were not different among the four groups Serum copper/albumin ratio increased in group III in comparison with group I (*p* < 0.05) Urinary copper concentration did not differ between groups I, II, and the control group, but its concentration was significantly higher in group III compared to other groups *p* < 0.001. Urinary cerulopasmin concentration significantly increased in group III in comparison with the other groups (*p* < 0.001), and it also increased in group II when compared with group I and the control group (*p* < 0.001). The copper/ceruloplasmin ratio in urine remarkably decreased in group III in comparison with the other groups (*p* < 0.001), and it also significantly decreased in group II when compared with group I and the control group (*p* < 0.001). The urinary copper/albumin ratio also decreased in group III compared to the other groups (*p* < 0.01) and decreased in group II in comparison with group I and control group (*p* < 0.001). Increase in urinary ceruloplasmin concentration correlated with urinary concentration of NAG (r = 0.846, *p* < 0.001)) and alfa-1-microglobulin (r = 0.608, *p* < 0.001) NAG and ·alfa-1-microglobulin concentrations in group II slightly increased in comparison with those in group I and control group (*p* < 0.05). NAG in group I was slightly higher than that in control group (*p* < 0.05). NAG and alfa- 1-microglobulin concentrations were clearly higher in group III than in the other groups (*p* < 0.001 and *p* < 0.01
	Talaei et al. [41] 2011 Cross-sectional study	42 TD2M patients with microalbuminuria	40 T2DM patients without microalbuminuria	Higher 24h urinary copper levels in microalbuminuria group compared to control group 36.14 μcg/L(14.54–57.74) vs. 14.77 μcg/L(10.17–19.37) *p* = 0.003 No significant difference between different subgroups based on HbA1C% levels
	Prabodh et al. [46] 2011 Case-control Study	40 patients with DKD Age 45–70 years	40 healthy subjects Age matched	No significant difference in serum copper levels between DKD group (165.42 ± 5.71 μg/dL) and control group (166.6 ± 5.48 μg/dL) (*p*> 0.05). No relation of Cu with microalbumin in DKD patients
	Stancic et al. [38] 2012 Case-Control Study	Hypertensive diabetic patients with or without renal insufficiency	Healthy subjects	Copper zinc superoxide dismutase activity was higher only in hypertensive diabetic with renal insufficiency
	Al Bayati et al. [40] 2015 Cross-sectional Study	Group I: 31 T2DM patients with microalbuminuria between 30 and 299 μg/mg Age 49.5 ± 7.6 years Group II: 29 T2DM patients with microalbuminuria below 30 μg/mg Age 52.2 ± 8.2 years	37 healthy subjects Age 48.9 ± 8.9 years	Group I showed a significant increase in urinary Cu/creatinine ratio compared with controls: 53.3 ± 3.2 vs. 44.2 ± 5.3 *p* < 0.05 No significant difference between Group II and controls in urinary Cu/creatinine ratio SOD was significantly decreased in group I compared to control group 30.6 ± 3.3 vs. 45 ± 6 *p* < 0.05 No significant difference between Group II and controls in SOD
	Hasamaki et al. [43] 2016 Cross-Sectional Study	149 T2DM patients Age 61.1 ± 17.6 years	206 non-diabetic patients	A high Zn/Cu ratio was associated with improved renal function levels (β = 0.137, *p* = 0.014) A high Zn/Cu ratio was associated with reduced risk of poor glycemic control in patients with type 2 diabetes, assessed by multivariate logistic regression analysis. (HbA1c ≥ 7%) (odds ratio = 0.382; 95% confidence interval, 0.165–0.884; *p* = 0.025)
	Takao et al. [44] 2022 Cross-sectional Study	651 patients with T2DM Age 65.1 ± 9.7 years	No control group	Diabetic kidney disease was identified in 220 patients A Higher Cu/Zn ratios is correlated with more frequent renal involvement Higher Cu levels in DKD patients compared to non DKD patients (100.5–15.5 vs. 97.0–15.6) *p* = 0.007 Higher Cu/Zn ratio in DKD patients compared to non DKD patients (1.247–0.265 vs. 1.155–0.242) *p* < 0.0001

## Abbreviations

CKD	Chronic Kidney Disease
Cu	Copper
DM	Diabetes Mellitus
DKD	Diabetic Kidney Disease
GFR	glomerular filtration rate
GDM	gestational diabetes mellitus
(HbA1c)	Glycosylated hemoglobin (HbA1c)
HOMA-β	homeostasis model assessment pancreatic beta cell function
HOMA-IR	homeostasis model assessment—insulin resistance
HOMA-S	Homeostasis model assessment—insulin sensitivity
LDL	low-density lipoprotein
Mg	Magnesium
Ors	Odds Ratio
O2	oxygen
OS	Oxidative stress
PAH	sodium p-aminoippurate
RCT	randomized controlled trial
ROS	reactive oxygen species
SMAD	homologous MAD (SMAD)
SOD	superoxide dismutase
STZ	streptozocin (STZ)
TGF-β	Transforming growth factor β
(TETA)	thethylenetetramine
TINag	antigen of tubulointerstitial nephritis (TINag)
Tm	maximal tubular concentration
TNF-α	tumor necrosis factor-alpha
T1DM	Type 1 diabetes mellitus
T2DM	type 2 diabetes mellitus
VDAC	selective voltage-dependent anion channel (VDAC)
Zn	Zinc
